# Distinct Expression/Function of Potassium and Chloride Channels Contributes to the Diverse Volume Regulation in Cortical Astrocytes of GFAP/EGFP Mice

**DOI:** 10.1371/journal.pone.0029725

**Published:** 2012-01-11

**Authors:** Jana Benesova, Vendula Rusnakova, Pavel Honsa, Helena Pivonkova, David Dzamba, Mikael Kubista, Miroslava Anderova

**Affiliations:** 1 Department of Cellular Neurophysiology, Institute of Experimental Medicine, Academy of Sciences of the Czech Republic, Prague, Czech Republic; 2 Laboratory of Gene Expression, Institute of Biotechnology, Academy of Sciences of the Czech Republic, Prague, Czech Republic; 3 Second Medical Faculty, Charles University, Prague, Czech Republic; 4 TATAA Biocenter, Gothenburg, Sweden; University of Louisville, United States of America

## Abstract

Recently, we have identified two astrocytic subpopulations in the cortex of GFAP-EGFP mice, in which the astrocytes are visualized by the enhanced green–fluorescent protein (EGFP) under the control of the human glial fibrillary acidic protein (GFAP) promotor. These astrocytic subpopulations, termed high response- (HR-) and low response- (LR-) astrocytes, differed in the extent of their swelling during oxygen-glucose deprivation (OGD). In the present study we focused on identifying the ion channels or transporters that might underlie the different capabilities of these two astrocytic subpopulations to regulate their volume during OGD. Using three-dimensional confocal morphometry, which enables quantification of the total astrocytic volume, the effects of selected inhibitors of K^+^ and Cl^−^ channels/transporters or glutamate transporters on astrocyte volume changes were determined during 20 minute-OGD *in situ*. The inhibition of volume regulated anion channels (VRACs) and two-pore domain potassium channels (K_2P_) highlighted their distinct contributions to volume regulation in HR-/LR-astrocytes. While the inhibition of VRACs or K_2P_ channels revealed their contribution to the swelling of HR-astrocytes, in LR-astrocytes they were both involved in anion/K^+^ effluxes. Additionally, the inhibition of Na^+^-K^+^-Cl^−^ co-transporters in HR-astrocytes led to a reduction of cell swelling, but it had no effect on LR-astrocyte volume. Moreover, employing real-time single-cell quantitative polymerase chain reaction (PCR), we characterized the expression profiles of EGFP-positive astrocytes with a focus on those ion channels and transporters participating in astrocyte swelling and volume regulation. The PCR data revealed the existence of two astrocytic subpopulations markedly differing in their gene expression levels for inwardly rectifying K^+^ channels (Kir4.1), K_2P_ channels (TREK-1 and TWIK-1) and Cl^−^ channels (ClC2). Thus, we propose that the diverse volume changes displayed by cortical astrocytes during OGD mainly result from their distinct expression patterns of ClC2 and K_2P_ channels.

## Introduction

The generation of brain edema, ultimately leading to an intracranial pressure increase and brain herniation, markedly alters the course and treatment of cerebral ischemia. Among brain cell types, astrocytes have been reported to contribute predominantly to edema formation. They support neuronal function and provide for the maintenance of ionic and neurotransmitter homeostasis under physiological conditions; however, they may also contribute to the worsening of brain damage under pathological conditions. The uptake of ions and amino acids may turn into an excessive osmolyte influx leading to the generation of edema, which in turn contributes to further ischemic damage. Several transporters and ion channels have been described as participating in astrocytic swelling (for a review, see [1]). Astrocytic Na^+^-dependent glutamate uptake, which is responsible for the termination of synaptic transmission and helps to protect neurons from the excitotoxic activity of glutamate, may reverse as a consequence of the ionic dis-balance accompanying energy depletion during severe ischemia and thus contribute to an increase of extracellular glutamate concentrations [2]. Additionally, K^+^-Cl^−^ (KCC) and Na^+^-K^+^-Cl^−^ (NKCC) co-transporters, the major regulators of neuronal and astrocytic Cl^−^ and Na^+^ gradients, have been found to participate in cell volume changes under pathological conditions. Increased NKCC activity has been shown to result in Na^+^, K^+^ and Cl^−^ accumulation, thus markedly contributing to astrocytic swelling [3], while KCCs, which are responsible for ion efflux, are rather involved in regulatory volume decrease (RVD) [4]. Nonetheless, a high extracellular concentration of K^+^ ([K^+^]_o_) may induce the reverse operation of KCCs and thus contribute to cell swelling, as described in retinal Müller cells [5]. Furthermore, the depolarization of the astrocytic membrane due to Na^+^/K^+^ ATP-ase inhibition results in excessive K^+^ and Cl^−^ influx via ion channels [6]. Despite the fact that a prominent role in K^+^ buffering has been suggested for the astrocytic Kir4.1 channel [7], [8], two-pore domain K^+^ (K_2P_) channels, especially TWIKs and TREKs, have been shown to be responsible for glial K^+^ uptake [9], and their contribution to glial volume homeostasis was described in Müller cells [10]. The activation of K_2P_ channels in response to ischemia has been demonstrated *in vitro* by Buckler and Honore [11] and the up-regulation of TREK-2 channels in conjunction with extracellular glutamate clearance was demonstrated in cultured rat astrocytes during anoxia/hypoglycemia by Kucheryavykh and co-authors [12]. Concurrently, astrocytic swelling is accompanied by RVD, which is also carried out by Cl^−^ and K^+^ channels [13]. The main anion channels responsible for Cl^−^ and organic osmolyte efflux are volume-regulated anion channels (VRACs), which play a predominant role in RVD. However, an anion efflux via VRACs has also been shown to contribute to further ischemic brain damage [14], [15] and moreover, VRACs have been reported to provide the anion influx that results in neuronal swelling [16].

In our recent *in situ* studies we have demonstrated that cortical astrocytes do not respond uniformly to hypoosmotic stress [17], [18] and that two differently responding groups of astrocytes – high response- (HR-) astrocytes and low response- (LR-) astrocytes – are present in the cortex of GFAP/EGFP mice [19] based on the volume changes evoked by oxygen-glucose deprivation (OGD) [20]. In the present study, we aimed to elucidate the basic mechanisms potentially underlying the different ability of the two astrocytic populations to regulate their volume in response to OGD. We have focused on the role of chloride and potassium channels/transporters and excitatory amino acid transporters in astrocytic swelling during OGD. For the quantification of cell volume, three-dimensional confocal morphometry has been employed. Moreover, we have used real-time, single-cell quantitative PCR (qPCR) to explore the gene expression profiles of cortical GFAP/EGFP astrocytes with a focus on those ion channels and transporters participating in astrocyte swelling and volume regulation. We have used a similar approach to that of Stahlberg and co-authors [21]; they have identified astrocytic subpopulations in the mouse brain using single-cell qPCR profiling.

## Materials and Methods

### Ethics Statement

All procedures involving the use of laboratory animals were performed in accordance with the European Communities Council Directive 24 November 1986 (86/609/EEC) and animal care guidelines approved by the Institute of Experimental Medicine ASCR Animal Care Committee on April 17, 2009; approval number 85/2009.

### Confocal 3D morphometry

#### Solutions

Artificial cerebrospinal fluid (ACSF) solution contained (in mM): NaCl 122.0, KCl 3.0, CaCl_2_ 1.5, MgCl_2_ 1.3, Na_2_HPO_4_ 1.25, NaHCO_3_ 28.0, D-glucose 10.0 (pH 7.4, in 95% O_2_/5% CO_2_). Osmolality was confirmed to be 300±5 mOsmol/kg with a vapor pressure osmometer (Vapro 5520, Wescor Inc., Logan, USA). Oxygen–glucose deprivation (OGD) was achieved by saturating glucose-free ACSF with 5% O_2_, 5% CO_2_ and 90% N_2_ (ACSF_OGD_). All inhibitors and their concentrations are listed in Table 1. DMSO, ethanol and methanol, which were used for the preparation of inhibitor stock solutions, did not affect astrocyte volume changes at their final concentrations (data not shown). The majority of chemicals, including inhibitors, were purchased from Sigma-Aldrich (St. Louis, MO, USA). DL-TBOA was purchased from Tocris Bioscience (Bristol, UK).

**Table 1:** List of used inhibitors.

	c (µM)	Targeted proteins in astrocytes
**Transporters**		
**DIOA**	100**	KCC [4]
[(dihydroindenyl)oxy]alkanoic acid		
**Bumetanide**	100**	NKCC [48]
3-(Aminosulfonyl)-5-(butylamino)-4-phenoxybenzoic acid		
**DL-TBOA**	100*	EAAT1, 2 [53]
DL-threo-b-benzyloxyaspartic acid		
**Cl^−^ channels**		
**NPPB**	100*	Cl^−^ channels, VRACs [24]
5-Nitro-2-(3-phenylpropylamino)benzoic acid		
**Tamoxifen**	30***	VRACs [24]
(Z)-1-(p-Dimethylaminoethoxyphenyl)-1,2-diphenyl-1-butene		
**DCPIB**	30*	VRACs [24]
4-(2-butyl-6,7-dichloro-2-cyclopentylindan-1-on-5-yl)oxybutyric acid		
**K^+^ channels**		
**BaCl_2_**	100	Kir channels [26]
**BaCl_2_**	1000	Kir, K_2P_ channels [27]
**Quinine**	200**	K2P channels [27]

KCC – K^+^-Cl^−^ co-transporter, NKCC – Na^+^-K^+^-Cl^−^ co-transporter, EAAT – excitatory amino acid transporter, VRAC – volume-regulated anion channel, K_2P_ – two-pore domain potassium channel, Kir - inwardly rectifying potassium channel.

Stock solutions were dissolved at 1,000× final concentration in DMSO (*); ethanol (**) or methanol (***). BaCl_2_ was dissolved at 1 mM concentration in double-distilled H_2_O (ddH_2_O).

#### Brain slice preparation

Quantification of astrocyte volume changes was carried out in acute brain slices of GFAP/EGFP mice [19]. Thirty to forty-day-old male/female mice were anesthetized with isoflurane followed by decapitation. Brains were dissected out and placed into ice-cold oxygenated ACSF. Transverse slices (400 µm) were cut using an HM650V vibratome (MICROM International GmbH, Waldorf, Germany). The slices were kept in continuously oxygenated ACSF, at room temperature (23–25°C), for at least 1 hour before starting experiments. To estimate the effect of each inhibitor on astrocytic swelling evoked by OGD, a single astrocyte was recorded in one slice and slices from 2–3 mice were used for each inhibitor.

#### Cell volume measurement and quantification

Changes in total astrocytic volume were measured and quantified using previously described method [17], [18], [20]. The slices were perfused with ACSF or ACSF_OGD_ (perfusion rate ∼5 ml/min). To examine the effect of inhibitors on astrocyte volume changes, they were applied during 40-minute OGD. All experiments were performed at room temperature (23–25°C). For cell volume quantification (Fig. S1) we have selected only brightly fluorescent cells at an approximate distance of ∼30 µm from the slice surface. The astrocytes were recorded as a set of two-dimensional (2D) sectional images with a resolution of 1024×1024 pixels using a Leica TCS SP system confocal microscope (Leica Germany) with a Leica 40× water immersion objective (0,8) HCX APO (Leica, Germany). EGFP was excited by an Ar laser set at 488 nm, and the emitted signal was recorded over the range of 510–552 nm using a TD488/543/633 filter. Each three-dimensional (3D) image of the cell was sectioned into 70–80 consecutive 2D images at a uniform spacing of 1 µm. Data were collected at 10 minute intervals. Image processing and morphometric measurements were performed using the program CellAnalyst developed at the Department of Cellular Neurophysiology, Institute of Experimental Medicine, Prague, Czech Republic [18].

#### Statistics

Changes in total astrocyte volume are presented as the mean ± SEM. Statistical significance was determined by a two-tailed unpaired Student's *t*-test. Differences between the groups were considered statistically significant when p<0.05, very significant p<0.01, and extremely significant p<0.001.

### Single-cell gene expression profiling

#### Preparation of cell suspensions from the cortex of GFAP/EGFP mice

GFAP/EGFP transgenic mice (30 days old) were deeply anesthetized with sodium pentobarbital (PTB, 100 mg/kg, i.p.), and perfused transcardially with cold (4–8°C) isolation buffer containing (in mM): NaCl 136.0, KCl 5.4, Hepes 10.0, glucose 5.5, osmolarity 290±3 mOsmol/kg. The forebrain was isolated by the removal of the olfactory lobes, cerebellum, and midbrain/hindbrain structures by dissection. To isolate the cerebral cortex, the brain was sliced in 1 mm coronal sections using a vibrating microtome HM650V (MICROM International GmbH, Walldorf, Germany), and the cerebral cortex was carefully dissected away from the ventral white matter tracks. The tissue was incubated with continuous shaking at 37°C for 90 minutes in 5 ml of a papain solution (20 U/ml) and 0.2 ml DNAase (both Worthington, Lakewood, NJ) prepared in isolation buffer. After papain treatment the tissue was mechanically dissociated by gentle trituration using a 1 ml pipette. Dissociated cells were layered on top of 5 ml of Ovomucoid inhibitor solution (Worthington) and harvested by centrifugation (140× g for 6 minutes). This method routinely yielded ∼1.5–2 million cells per mouse forebrain.

#### Collection of single EGFP-positive cells

Dissociated cells were sorted using flow cytometry (BD FACSCalibur); they were kept on ice throughout the sorting procedure. Cell aggregates were removed by filtering with a 30 nm cell strainer (Becton Dickinson, USA). The flow cytometry instrument was manually calibrated to deposit single cells in the centre of each collection tube. Propidium iodide was added to the suspension of cells for checking viability. Single cells were collected into 96-well plates (Life Technologies, Czech Rep.) containing 5 µl nuclease-free water with bovine serum albumin (1 mg/µl, Fermentas, Biogen, Czech Rep.) and 20 U RNAseOut (Life Technologies, Czech Rep.) per well. The plates were then placed on a pre-cooled rack. The astrocytes were collected based upon their positivity for EGFP and their viability. Plates with collected cells were immediately frozen at −80°C.

#### cDNA synthesis

A modified protocol of A. Stahlberg [21] was used for cDNA synthesis. SuperScript III RT (Life Technologies, Czech Rep.) was used for reverse transcription. Lysed single cells in 5 µl water containing 0.5 mM dNTP (Promega, Germany), 1.0 mM oligo(dT15) (Invitrogen) and 1.0 mM random hexamers (Invitrogen) were incubated at 70°C for 5 min. 50 mM Tris–HCl, 75 mM KCl, 3 mM MgCl_2_, 5 mM dithiothreitol, 20 U RNaseOut and 100 U SuperScript III (all Invitrogen; final concentrations) were added to a final volume of 10 µl. Reverse transcription was performed at 25°C for 5 min, 50°C for 60 min, 55°C for 10 min and terminated by heating to 70°C for 15 min. All samples were diluted to 40 µl with water before qPCR.

#### Primer design and optimization of assays

Primers were designed using BeaconDesigner software (version 7.91, Premier Biosoft International). All primers except those for *Cspg4* and *Nkcc1* were designed to span an intron to avoid amplification of genomic DNA. BLAST (Basic Local Alignment Search Tool) searches revealed no pseudogenes. The primer sequences are shown in Table S1. All assays of single cells were optimized so as to not generate primer dimers before cycle 45, to have a PCR efficiency of at least 90%, and to amplify all known splice forms documented by the National Center for Biotechnology Information (NCBI). Calibration curves with purified PCR products (QIAquick PCR Purification Kit; Qiagen, Germany) were used to establish the linearity of the assays. The formation of the correct PCR products was confirmed by electrophoresis on 20 g/L agarose gels for all assays and by melting-curve analysis of all samples. Five individual cells per assay were tested, and no genomic DNA amplification was observed.

#### qPCR

A Biorad CFX384 (Biorad, Czech Rep.) was used for all qPCR measurements. To each reaction (10 µl) containing iQ SYBR Green Supermix (BioRad) and 300 nM of each primer (EastPort, Czech Rep.), we added 3 µl diluted cDNA. The temperature profile was 95°C for 3 min followed by 50 cycles of amplification (95°C for 15 s, 60°C for 15 s and 72°C for 20 s). All samples were analyzed by melting curve analysis.

#### Data processing and statistics

For all assays the limit of detection (LOD) was assumed to be the highest Cq (quantification cycle) value measured reflecting the expected product based on melt curve analysis. All Cq values that were above the limit of detection and hence were reflecting the formation of aberrant products, and any missing data, were replaced by the Cq at the limit of detection +1. The correction was performed separately for each gene and effectively corresponds to assigning a concentration to the off-scale measurements that is 50% of the concentration we reliably detect. Cq values were converted to relative quantities and transformed to a log scale [22].

Dendrograms, principal component analysis (PCA) and Kohonen self-organizing maps were calculated using GenEx software (ver. 5.3, MultiD, Sweden). The expression of each gene was mean-centred for profiling analysis with multivariate methods. Dendrograms were calculated using Ward's algorithm and Euclidean distance measure. Spearman correlation coefficients were calculated for all gene pairs. Because of the significant number of off-scale data that were corrected, the distribution of data was not necessarily Gaussian, and non-parametric statistical tests were used to assess significance. The expression levels measured in groups of cells were compared using the non-parametric Kruskal-Wallis test with Dunn's post-test. For Kohonen self-organizing maps (SOMs) and potential curve analysis, autoscaled expression values were used to give all genes equal weight in the clustering algorithms. The parameters used for the Kohonen SOMs were: 3x1 map, 0.10 learning rate, 2–3 neighbors and 10000 iterations. The calculated clusters did not depend on the parameters' settings.

## Results

Astrocyte heterogeneity has been described previously as morphological differences, distinct functional properties and variations in the expression of receptors, ion channels and transporters [23]. In our previous study [20] we described two astrocytic subpopulations in the cortex of GFAP/EGFP mice differing in their volume changes induced by OGD. One subpopulation, referred to as high response-astrocytes (HR-astrocytes), markedly increased their total cell volume when exposed to OGD, while only small volume changes were evoked in the second subpopulation, referred to as low response-astrocytes (LR-astrocytes). Both subpopulations were morphologically identical, displayed passive currents and expressed EAAT1 and EAAT2 transporters. Here, we focus on elucidating the differences between these two subpopulations that might originate from the diverse functioning/expression of ion channels and transporters that are involved in astrocytic swelling and cell volume regulation. We have employed a wide range of transporter- and ion channel-inhibitors (Table 1) to study their effects on astrocyte volume changes *in situ* evoked by OGD.

### Astrocyte swelling evoked by oxygen-glucose deprivation

**Figure 1:**
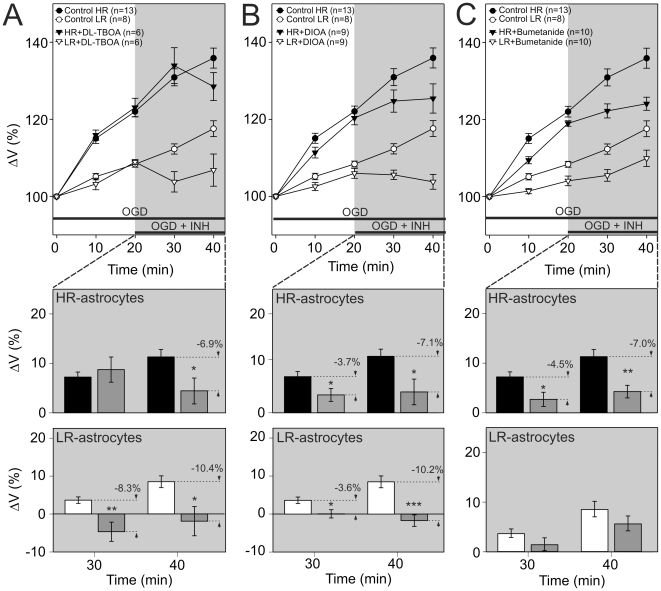
Inhibitors of glutamate transporters and K^+^-Cl^−^ co-transporters reduce the OGD-induced swelling in both astrocytic subpopulations. The effect of 100 µM DL-TBOA, an inhibitor of excitatory amino acid transporters (**A**), 100 µM DIOA, an inhibitor of K^+^-Cl^−^ co-transporter (**B**), and 100 µM bumetanide, an inhibitor of Na^+^-K^+^-Cl^−^ co-transporter (**C**). **A–C top:** Time course of volume changes in HR-astrocytes (filled circles) and LR-astrocytes (empty circles) during 40-minute OGD (control) and during 20-minute OGD followed by 20-minute OGD with the application of an inhibitor in HR-/LR-astrocytes (filled/empty triangles). **A–C bottom:** The effect of inhibitors was evaluated in each individual cell and expressed as the percent cell volume increase/decrease related to the maximal volume after 20-minute OGD, which was set as 0%. Note that the application of DL-TBOA and DIOA led to a swelling reduction in both HR- and LR- astrocytes, while bumetanide only affected the swelling in HR-astrocytes. Asterisks indicate significant differences from controls (*p*<0.05 (*, significant), *p*<0.01 (**, very significant), *p*<0.001 (***, extremely significant)).

**Figure 2:**
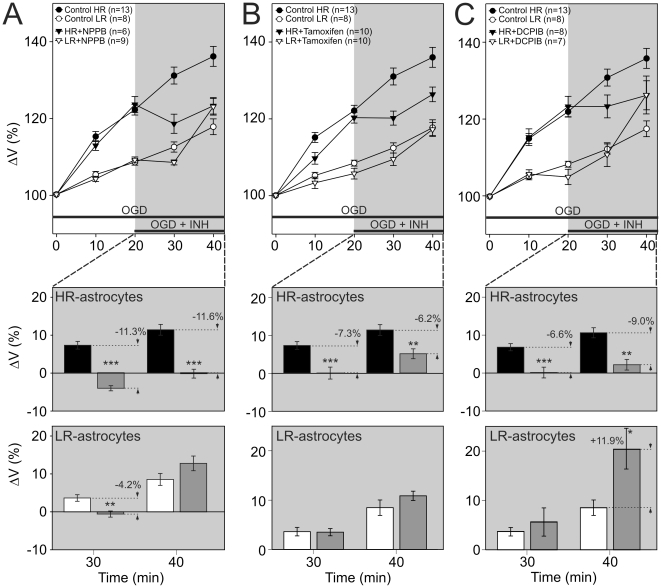
Inhibitors of Cl^−^ channels differently affect OGD-induced swelling in HR-and LR-astrocytes. The effect of 100 µM NPPB, a non-specific inhibitor of chloride channels (**A**), 30 µM Tamoxifen (**B**) and 30 µM DCPIB (**C**), inhibitors of volume regulated anion channels. **A–C top:** Time course of volume changes in HR-astrocytes (filled circles) and LR-astrocytes (empty circles) during 40-minute OGD (control) and during 20-minute OGD followed by 20-minute co-application of ACSF_OGD_ plus an inhibitor in HR-/LR-astrocytes (filled/empty triangles). **A–C bottom:** The effect of the inhibitors was evaluated in each individual cell and expressed as the percent cell volume increase/decrease related to the maximal volume after 20-minute OGD, which was set as 0%. Note that in HR-astrocytes the application of VRAC inhibitors (Tamoxifen and DCPIB) reduced the swelling induced by OGD, while in LR-astrocytes the application of DCPIB resulted in an additional swelling. Asterisks indicate significant differences from controls (*p*<0.05 (*, significant), *p*<0.01 (**, very significant), *p*<0.001 (***, extremely significant)).

To examine the effect of inhibitors on acute astrocytic swelling, brain slices were exposed to 40-minute OGD. The values of total astrocyte volume obtained during 40-minute OGD in the absence of any inhibitor were used as control values and compared to those obtained in the presence of inhibitors. The astrocyte volume at t = 0 was set to 100%, and the volume changes were expressed relative to this baseline as an increase in percentage. HR- and LR-astrocytes were classified using the same criteria as described previously [20]. Briefly, if the total cell volume increase was less than 10% (LR-astrocytes), the relative changes in total volume, cell soma volume and the volume of the processes were similar. If the total cell volume increase was greater than 10% (HR-astrocytes), the swelling of the cell processes exceeded the swelling of the soma. The total cell volume (V) of HR-astrocytes (n = 13) increased to 122.1±1.4% after 20 minutes and to 135.9±2.6% after 40 minutes of OGD. LR-astrocytes (n = 8) increased their volume to 108.4±0.8% after 20 minutes and to 117.6±2.1% after 40 minutes of OGD (Fig. 1, 2, 3 top). The effects of inhibitors were studied as follows: brain slices were first exposed to OGD alone for 20 minutes, followed by a 20 minute application of ACSF_OGD_ together with an inhibitor (Fig. S2). Using this approach, an individual cell could be classified as either an HR- or LR- astrocyte based on its volume changes during the first 20 minutes. In order to evaluate the effect of inhibitors on astrocytic swelling, volume changes were evaluated in each cell individually and expressed as a percent cell volume increase/decrease related to the volume obtained after the first 20 minutes of OGD, which was set as 0% (Fig. 1, 2, 3 bottom).

**Figure 3:**
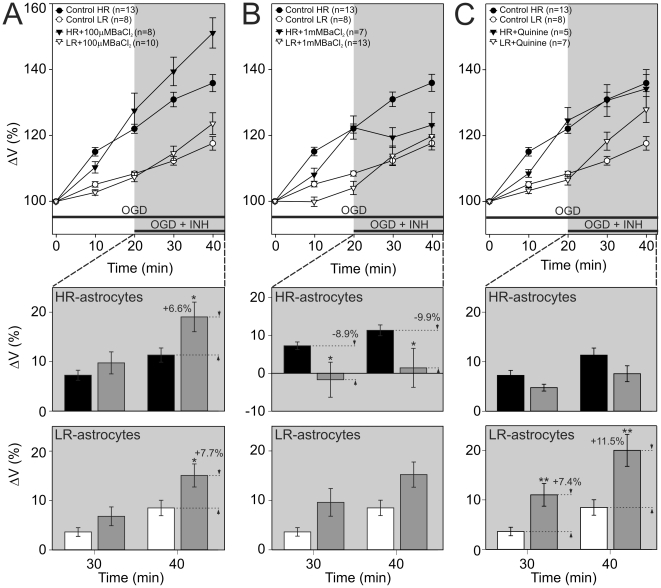
Inhibitors of K^+^ channels have an opposite effect on HR-/LR-astrocytes when applied during OGD. The effect of 100 µM BaCl_2_ (**A**), an inhibitor of inwardly rectifying potassium channels, 1 mM BaCl_2_ (**B**) and 200 µM Quinine (**C**), inhibitors of two-pore domain potassium channels. **A–C top:** Time course of volume changes in HR-astrocytes (filled circles) and LR-astrocytes (empty circles) during 40-minute OGD (control) and during 20-minute OGD followed by 20-minute co-application of ACSF_OGD_ plus an inhibitor in HR-/LR-astrocytes (filled/empty triangles). **A–C bottom:** The effect of inhibitors was evaluated in each individual cell and expressed as the percent cell volume increase/decrease related to the maximal volume after 20-minute OGD, which was set as 0%. Note that the application of 1 mM BaCl_2_ or Quinine inhibited swelling in HR-astrocytes, while in LR-astrocytes it had the opposite effect. In contrast, the application of 100 µM BaCl_2_ had the same effect in both groups of astrocytes. Asterisks indicate significant differences from controls (*p*<0.05 (*, significant), *p*<0.01 (**, very significant), *p*<0.001 (***, extremely significant)).

### Inhibition of glutamate and K^+^-Cl^−^ transporters reduces the astrocytic swelling induced by OGD in both subpopulations, while inhibition of Na^+^-K^+^-Cl^−^ co-transporters affects only HR-astrocytes

The disruption of ionic and excitatory amino acid (EAA) homeostasis that occurs during cerebral ischemia results in enhanced glutamate/K^+^ uptake by astrocytic transporters, leading to marked volume changes in astrocytes during ischemia [1]. In order to determine whether these transporters underlie the differences in volume changes between HR-/LR-astrocytes, we tested the effect of DL-TBOA, an inhibitor of the excitatory amino acid transporters EAAT1 and EAAT2, bumetanide, an inhibitor of Na^+^-K^+^-Cl^−^ co-transporters (NKCCs), and DIOA, an inhibitor of K^+^-Cl^−^ co-transporters (KCCs), on the astrocyte swelling evoked by OGD (for concentrations see Table 1). The application of DL-TBOA led to a significant volume decrease in both astrocytic subpopulations (Fig. 1A); however, the course of inhibition was slightly different. While HR-astrocyte swelling remained unaffected during the first 10 minutes, it decreased by 6.9% after 20 minutes of ACSF_OGD_ and DL-TBOA co-application (n = 6). In LR-astrocytes swelling was reduced already after 10 minutes, and after 20 minutes the swelling was reduced by 10.4% (n = 6). Similarly, the inhibitor of KCCs - DIOA - inhibited cell swelling in both astrocyte groups (Fig. 1B). A 20 minute application of ACSF_OGD_ and DIOA reduced cell swelling by 7.1% in HR-astrocytes (n = 9) and by 10.2% in LR-astrocytes (n = 9). On the other hand, the inhibition of NKCCs by bumetanide led to the reduction of swelling in HR-astrocytes (by 7.0% after 20 minute ACSF_OGD_ and bumetanide co-application; n = 10), while in LR-astrocytes the application of bumetanide did not significantly alter the extent of cell swelling (n = 10; Fig. 1C). In summary, the ion influx via NKCCs significantly contributed to HR-astrocyte swelling during OGD, while it was not involved in the volume changes observed in LR-astrocytes.

### Inhibition of volume-regulated anion channels or two-pore domain K^+^ channels during OGD reveals differences between HR- and LR-astrocytes

Since in many cell types, including astrocytes, VRACs are the predominant channels activated by swelling [13], we have examined the effects of three VRAC inhibitors – NPPB, tamoxifen and DCPIB (for concentrations see Table 1). A non-selective inhibitor of anion channels, NPPB had an opposite effect on the two astrocytic subpopulations (Fig. 2A). The swelling of HR-astrocytes was decreased by 11.6% after 20 minutes of ACSF_OGD_ and NPPB co-application (n = 6), while in LR-astrocytes the swelling was first reduced by 4.2% after 10 minutes and then it rather increased (n = 9). Similarly, tamoxifen and DCPIB also had a diverse effect on HR-/LR-astrocytes. Tamoxifen reduced the HR-astrocyte swelling by 6.2% after 20 minutes of its application (n = 10) and the DCPIB application led to the swelling reduction of 9.0% after 20 minutes (n = 8). On the other hand, the LR-astrocyte swelling was not appreciably affected by Tamoxifen (Fig. 2B), whereas DCPIB application led to their additional swelling (Fig. 2C). Moreover, the block of HR-astrocyte swelling by NPPB was more effective than that induced by the other inhibitors, which is in accordance with the lower specificity of the inhibitor as reported previously by Abdullaev and co-authors [24], suggesting that more types of chloride channels are involved in HR-astrocyte swelling. In summary, DCPIB, which is currently the most selective inhibitor of VRACs known, reduced the swelling induced by OGD in HR-astrocytes, while in LR-astrocytes this inhibitor caused their additional swelling.

In view of previous findings that the main K^+^ channels expressed in mature astrocytes are Kir4.1 and two-pore domain channels [25], [26], we have examined the effect of BaCl_2_ and quinine (for concentrations see Table 1) on the volume changes of HR-/LR-astrocytes during OGD. The application of 100 µM BaCl_2_, which has been shown to preferentially inhibit Kir channels [26], led to an additional swelling after 20 minutes of OGD in both subpopulations of astrocytes. The swelling increased by 6.6% in HR-astrocytes (n = 8) and by 7.7% in LR-astrocytes (n = 10; Fig. 3A). Conversely, 1 mM BaCl_2_, used as a blocker of both Kir and K_2P_ channels [27], completely inhibited the swelling of HR-astrocytes (9.9% reduction after 20 minutes; n = 7), while in LR-astrocytes it rather led to an additional swelling (not significant, n = 13; Fig. 3B). Equally, the application of quinine slightly reduced the swelling of HR-astrocytes (not significant; n = 5), while markedly increased the swelling of LR-astrocytes by 11.5% after 20 minutes (n = 7; Fig. 3C). In conclusion, the inhibition of Kir channels revealed that they contribute predominantly to the efflux of K^+^ in both astrocytic subpopulations. On the other hand, the inhibition of K_2P_ channels disclosed their participation in K^+^ uptake in HR-astrocytes, while in LR-astrocytes they rather contribute to K^+^ efflux.

### The presence of two distinct populations is not linked to gender or location in the cortical layers

Since the sensitivity of astrocytes to OGD might be influenced by their location within the CNS, we quantified the incidence of HR-/LR- astrocytes based on their position in the cortical layers. The distribution of astrocytes between the two groups in the different cortical layers was as follows: I layer – 46% HR, 54% LR (n = 46), II–III layers – 41% HR, 59% LR (n = 113), IV–VI layers – 48% HR, 52% LR (n = 133). Although females have been shown to be more resistant to ischemic injury than males in clinical studies as well as in animal models [28], gender does not underlie the existence of two astrocytic groups with a different sensitivity to ischemic conditions. In females, 45% of measured cells were classified as HR- and 55% as LR-astrocytes (total number of cells n = 193); similarly in males, 49% of cells belonged to HR- and 51% to LR- astrocytes (n = 168). In conclusion, neither the location of astrocytes in the cortical layers, nor the gender of the mice underlies the presence of two astrocytic populations differing in their response to OGD (Fig. S3).

### Single-cell gene expression profiling reveals astrocyte heterogeneity in the cortex of GFAP/EGFP mice

**Figure 4:**
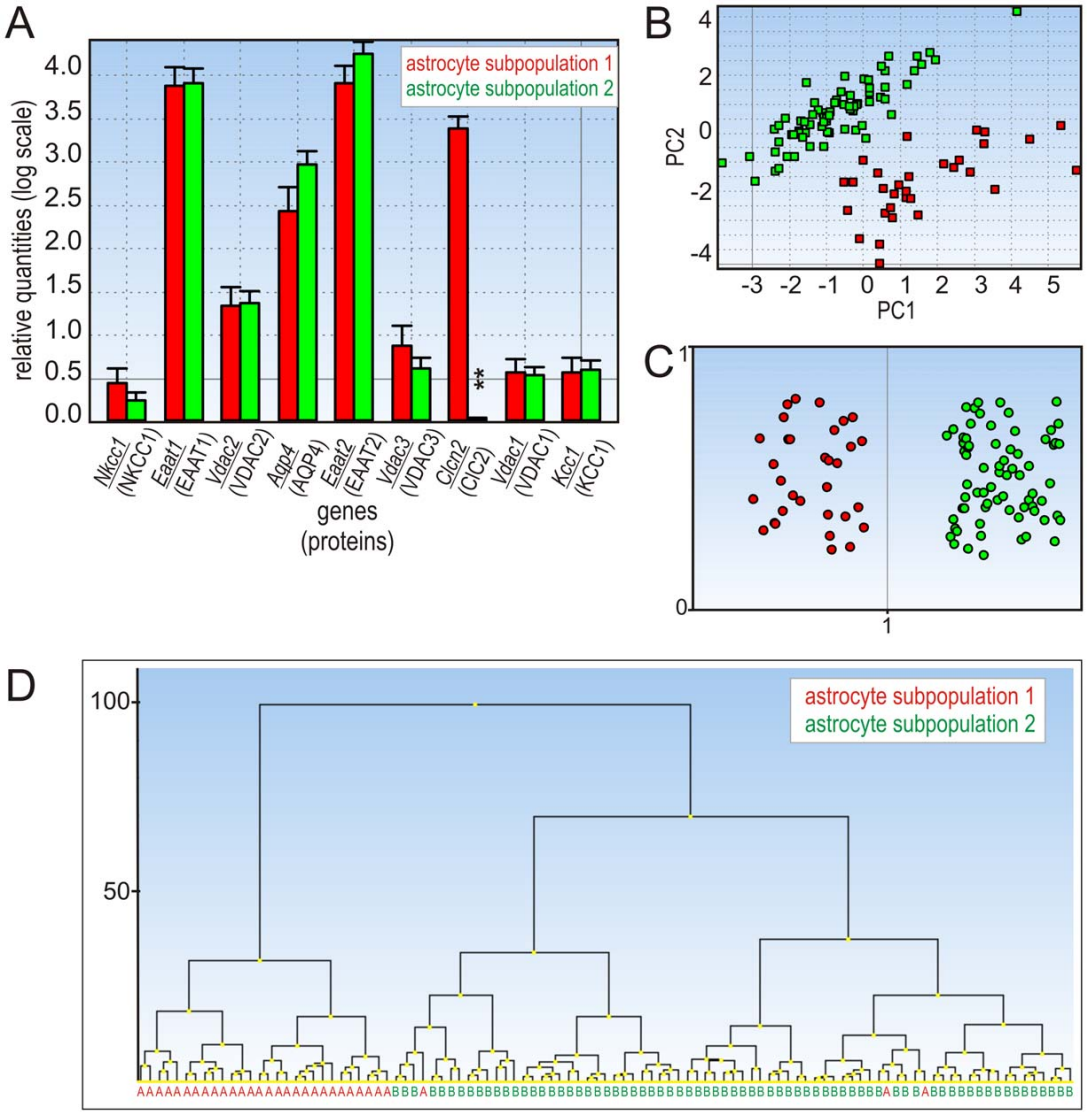
Experiment I: gene expression profiling of distinct astrocytic subpopulations. **A:** Bar plot with SEM for all the expressed genes; significant differences are indicated with asterisks (*p*<0.05 (*), *p*<0.01 (**), *p*<0.001 (***). **B:** Principal component analysis. The identification of 2 astrocytic subpopulations is along the first principal component, which accounts for most of the variation in the measured data. **C:** Clustering of astrocytes using Kohonen SOMs. The expression levels of all genes were mean-centered. Each dot represents one cell. **D:** Dendrogram based on all astrocytic genes. The y-axis shows the distance between groups.

**Figure 5:**
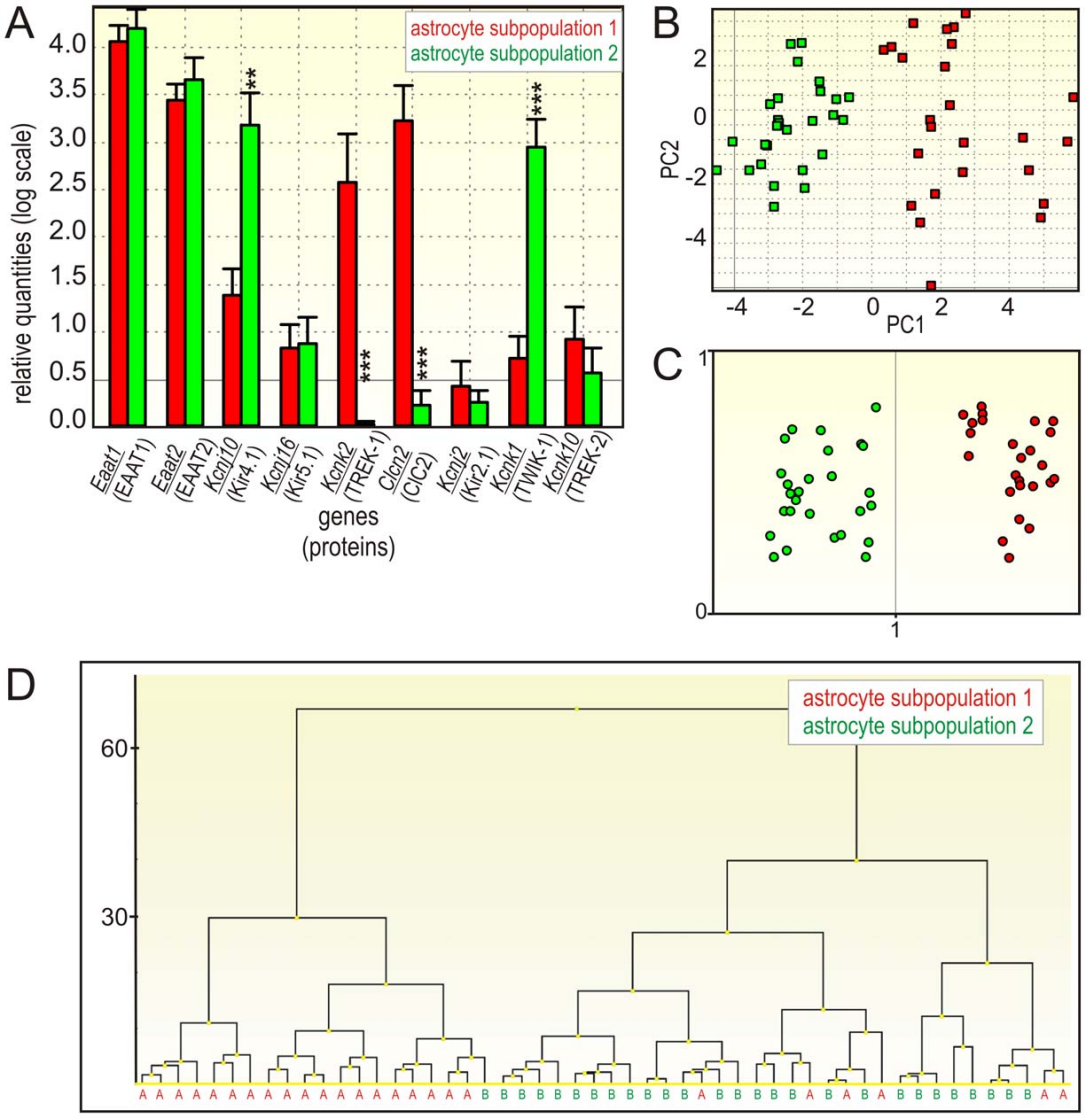
Experiment II: gene expression profiling of distinct astrocytic subpopulations. **A:** Bar plot with SEM for all the expressed genes; significant differences are indicated with asterisks (*p*<0.05 (*), *p*<0.01 (**), *p*<0.001 (***). **B:** Principal component analysis. The identification of 2 astrocytic subpopulations is along the first principal component, which accounts for most of the variation in the measured data. **C:** Clustering of astrocytes using Kohonen SOMs. The expression levels of all genes were mean-centered. Each dot represents one cell. **D:** Dendrogram based on all astrocytic genes. The y-axis shows the distance between groups.

We have also considered that the different abilities of astrocytes to regulate their volume might originate from distinct cellular phenotypes characterized by ion channel/transporter expression profiles. Based on pharmacological analyses, we have selected genes encoding astrocytic channels and transporters that could be responsible for the divergent volume regulation in cortical astrocyte subpopulations and performed quantitative single-cell PCR analyses. EGFP-positive cells were collected using fluorescence-activated cell sorting (FACS). Collected brightly fluorescent astrocytes expressed high mRNA levels of *Eaat1*, *Eaat2* and *Aqp4*, which code the typical astrocytic markers: EAAT transporters 1 and 2 and AQP4 water channels [29]. EGFP-positive cells expressing genes typical of NG2 glia, namely *Pdgfr* and *Cspg4* (coding PDGFα receptors and chondroitin sulfate proteoglycan 4), were excluded from the analyses. The first round of screening, in which all genes listed in Table S1 were tested, revealed significant differences in gene expression among cortical astrocytes. For further analyses, we have chosen 16 genes (Table S1) that showed marked differences in their expression levels during the first round of screening. The expression profiling was performed in two independent experiments (I and II). In experiment I, the expression profiles of *Nkcc1*, *Eaat1-2*, *Vdac1-3*, *Aqp4*, *Clcn2* and *Kcc1* were determined in 103 astrocytes. The numbers of astrocytes positive for individual genes are listed in Table S2. At first, we looked for correlations between the genes using Spearman's correlation. The correlation coefficient is a value between −1 and 1, where 1 reflects a perfect positive correlation, −1 reflects a perfect negative correlation and 0 indicates no correlation. Significant positive correlations were found between *Eaat1 – Eaat2*, *Eaat1 – Aqp4*, *Eaat1 – Vdac1*, *2*, *3*, *Eaat2 – Aqp4* and *Eaat2 – Vdac2*. The correlation coefficients for genes tested in experiment I are listed in Table S4. We have employed three independent analyses: principal component analysis (PCA) that enables the visualization of multidimensional data in a two- or three-dimensional plot [30], hierarchical clustering and Kohonen SOMs (Fig. 4 and 5). Based on these three independent mathematical algorithms, we have identified two subpopulations of astrocytes – subpopulation 1 and subpopulation 2 – that differ in their gene expression profiles. In experiment I (Fig. 4), both subpopulations expressed comparable levels of *Eaat1-2*, *Aqp4*, *Nkcc1*, *Kcc1* and *Vdac1-3*; however, in subpopulation 2 a markedly reduced expression of Clcn2 was detected. In experiment II, the expression of *Eaat1-2*, *Kcnj2*, *10*, *16*, *Kcnk1*, *2*, *10* and *Clcn2* was measured in 52 individual astrocytes. The numbers of astrocytes positive for individual genes after dividing the cells into two astrocytic subpopulations are listed in Table S3. In order to compare mRNA levels from independent experiments and to follow the same astrocytic subpopulations that were identified in experiment I, we have analyzed *Eaat1*, *Eaat2* and *Clcn2* in both experiments. Significant positive correlations were found between *Eaat1 – Eaat2* and *Eaat2 – Kcnj10*. Interestingly, significant negative correlations were found between *Kcnk2 – Kcnk1* and *Clcn2 – Kcnk1*, which means that the majority of cells expressing *Kcnk1* (the gene encoding TWIK-1) do not express *Kcnk2* and *Clcn2* (encoding TREK-1 and ClC2 channels). The Spearman correlation coefficients for the genes tested in experiment II are listed in Table S5. In experiment II (Fig. 5), in addition to *Clcn2*, *Kcnj10*, *Kcnk1* and *Kcnk2* were also identified as genes separating the two astrocytic subpopulations. While *Kcnj10* and *Kcnk1* were highly expressed in subpopulation 2, their expression in subpopulations 1 was significantly lower. On the other hand, *Kcnk2* was highly expressed in subpopulation 1, while it was almost absent in subpopulation 2.

In summary, we found two astrocytic subpopulations in the cortex of GFAP/EGFP mice related to distinct expression profiles of *Clcn2*, *Kcnj10* and *Kcnk1, 2* (genes encoding ClC2, Kir4.1, TWIK-1 and TREK-1).

## Discussion

**Figure 6:**
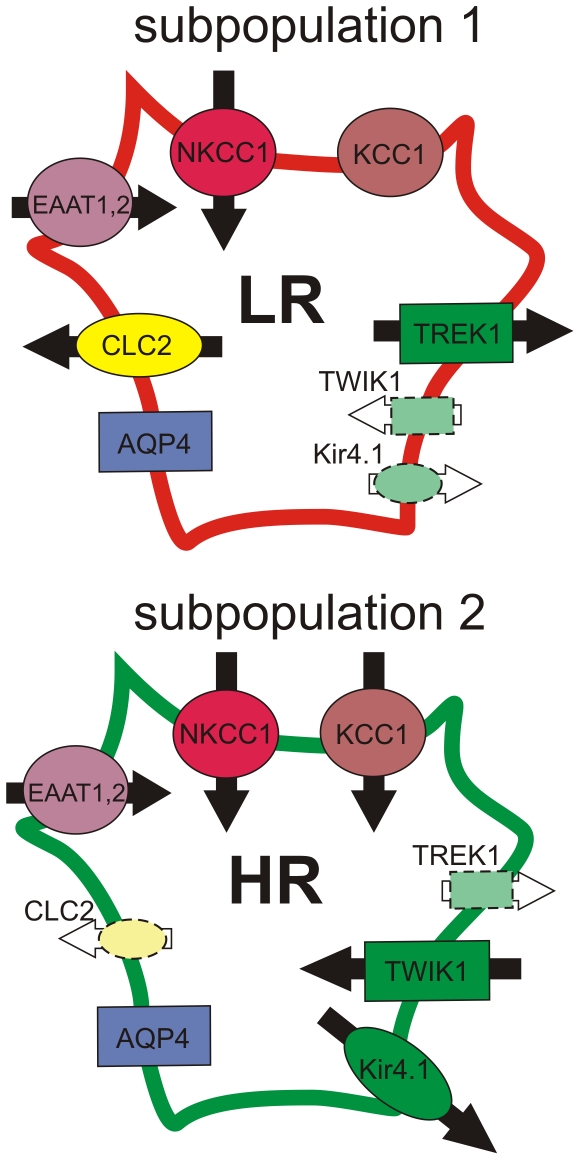
Two astrocytic subpopulations differing in the gene expression levels of K^+^/Cl^−^ channels. We propose a correlation of subpopulation 1 with LR-astrocytes and subpopulation 2 with HR-astrocytes due to the diverse gene expression levels for ClC2 and TREK-1, which are responsible for K^+^ and Cl^−^ efflux and thus contribute to cell volume regulation. Ion channels outlined with dashed line indicate their low gene expression levels. Arrows indicate the proposed direction of ion/EAA fluxes through the channels/transporters during OGD based on the effect of the relevant inhibitors. AQP4 – aquaporin channel (subtype AQP4), ClC2 - chloride channel (subtype ClC2), EAAT – excitatory amino acid transporter (subtypes EAAT1 and EAAT2), HR – high response astrocyte, TWIK1 and TREK1 – two-pore domain potassium channels (subtypes TWIK-1, TREK-1), KCC1 – K^+^-Cl^−^ co-transporter (subtype KCC1), Kir4.1 – inwardly rectifying potassium channel, LR – low response astrocyte, NKCC1 – Na^+^-K^+^-Cl^−^ co-transporter (subtype NKCC1).

In the present study we have demonstrated that the two previously described cortical astrocytic populations – HR- and LR-astrocytes – respond differently to Cl^−^ and K^+^ channel inhibitors when applied during OGD. In accordance with the pharmacological experiments, quantitative single-cell PCR profiling revealed that cortical astrocytes are heterogeneous with respect to the gene expression profiles of the ion channels/transporters that participate in their volume regulation. We distinguished subpopulation 1, which is characterized by high gene expression levels of Cl^−^ channels and K^+^ channels, namely ClC2 and TREK-1, and subpopulation 2, displaying high gene expression levels of K^+^ channels – Kir4.1, TWIK-1. As we hypothesize that the diverse HR- and LR-astrocyte swelling might be a consequence of different gene expression levels for ClC2 and TREK-1, both contributing strongly to astrocyte volume regulation, we propose a correlation of subpopulation 1 with LR-astrocytes and subpopulation 2 with HR-astrocytes. Their proposed correlation with the HR-/LR- classification scheme is summarized in Figure 6.

**Figure 7:**
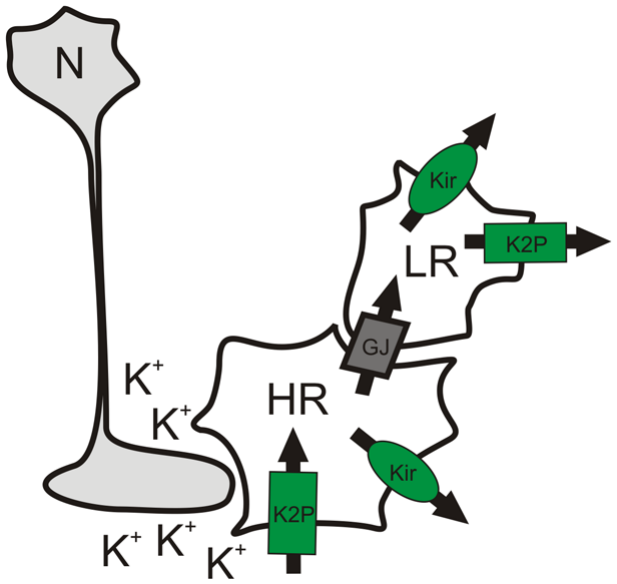
Proposed K^+^ movement from HR- to LR-astrocytes during OGD. HR-astrocytes exposed to higher [K^+^]_o_ take up K^+^ (by K_2P_ channels or co-transporters), which is either released via Kir channels or redistributed through the astrocytic syncytium and then extruded by Kir and K_2P_ channels in LR-astrocytes. HR – high response astrocyte, LR – low response astrocyte, N – neuron, K2P – two-pore domain potassium channel, Kir – inwardly rectifying potassium channel, GJ – gap junction.

The most striking dissimilarity between HR- and LR- astrocytes was found during the inhibition of K_2P_ channels by 1 mM BaCl_2_ and 200 µM quinine. Barium chloride (1 mM), which was shown to block the inwardly rectifying K^+^ channel TWIK-1 [27], markedly reduced the swelling of HR-astrocytes, thus suggesting that TWIK-1 channels contribute to K^+^ influx into HR-astrocytes. In contrast, quinine, which inhibits the outwardly rectifying K_2P_ channel TREK-1, increased the swelling of LR-astrocytes, thus implying that K_2P_ channels provide K^+^ extrusion in LR-astrocytes. Since polymodal TREK channels are activated by mechanical stress/cell swelling, their role in RVD is highly possible [31]. These data are also in agreement with previous studies of Skatchkov and co-authors [10] in retinal Müller glia. Moreover, our quantitative single-cell PCR analysis revealed distinct astrocytic subpopulations: subpopulation 1 expressing extremely high levels of *Kcnk2* (a gene for TREK-1) and subpopulation 2 expressing high levels of *Kcnk1* (a gene for TWIK-1). Combining our pharmacological and PCR data, we propose that TREK-1 channels are uniquely expressed in LR-astrocytes and thus contribute to their efficient cell volume regulation during OGD by providing K^+^ efflux. On the other hand, HR-astrocytes strongly express TWIK-1, which is responsible for enhanced K^+^ uptake and their swelling. A similar heterogeneity in K_2P_ channel expression was demonstrated in hippocampal astrocytes [26]. Unequal TWIK-1/TREK-1 expression levels might originate from the local environment of each astrocyte in the cortex, as proposed in Fig. 7. We hypothesize that HR-astrocytes comprise cells locally exposed to a high extracellular K^+^ concentration ([K^+^]_o_), thus predominantly expressing inwardly rectifying channels for effective K^+^ uptake. LR-astrocytes, in contrast, might receive K^+^ as a consequence of spatial buffering and release it into the ECS. Apparently, K_2P_ channels play an important role during ischemia as demonstrated by Kucheryavykh and co-authors [12], who showed the involvement of TREK-2 channels in response to anoxia/hypoglycemia in cultured rat astrocytes. Our present findings demonstrate the involvement of TREK-1 rather than TREK-2 in astrocytic swelling during OGD; however, such discrepancies might originate from the different properties of cultured astrocytes isolated from 1-day-old rats [12] and those in brain slices of 30-day-old mice, and moreover, from employing different models of brain injury. Nonetheless, in our recent work, Pivonkova and co-authors [25] described the increased expression of TREK-1 in hippocampal astrocytes in the CA1 region following global cerebral ischemia *in vivo*.

Furthermore, we have demonstrated that in HR- and LR-astrocytes the inhibition of Kir channels led to additional cell swelling, which suggests that Kir channels contribute rather to K^+^ efflux than K^+^ uptake after 40 minute OGD. This is in line with data demonstrating the participation of Kir4.1 in RVD [32], [33], [34], [35]. In contrast to the comparable effect of a Kir channel inhibitor (100 µM BaCl_2_) on the extent of cell swelling in both astrocytic subpopulations, we found a significantly increased expression of *Kcnj10* (encoding Kir4.1) in subpopulation 2 that comprises HR-astrocytes. In general, an astrocyte is considered as a multifunctional unit in which the role of Kir channels can differ in the various plasma membrane regions of a given cell, providing K^+^ uptake at sites facing the synapses and K^+^ release from astrocyte endfeet that contact blood vessels [36]. Based on this assumption, changes in total astrocytic volume thus reflect both K^+^ uptake as well as K^+^ release. As blocking Kir4.1 channels by 100 µM barium resulted in a similar effect on LR- and HR-astrocyte volume, we suggest that in both types of astrocytes the proportion of Kir4.1 channels that provide K^+^ uptake (causing astrocytic swelling) and those providing K^+^ release (causing astrocytic shrinkage) are comparable. This might ultimately lead to the similar impact of Kir4.1 inhibition on astrocyte volume despite the observed differences in *Kcnj10* expression between the two subpopulations. We hypothesize that the high expression of *Kcnj10* in HR-astrocytes may mirror the increased requirements for Kir4.1 channel densities on HR-astrocyte membranes as these cells might be exposed to higher local extracellular concentration of K^+^ than are LR-astrocytes, thus providing significantly greater K^+^ uptake/K^+^ release. This accords well with our previous findings, in which we described the presence of cortical astrocytes differing in Kir4.1 levels based on immunohistochemistry [20]. Nonetheless, a detailed analysis of different cellular compartments, such as astrocytic processes in the vicinity of neuronal synapses or those neighboring blood vessels, might elucidate the relative contribution of K^+^ influx and efflux to astrocyte volume with respect to the local uptake/release provided by Kir4.1 channels. Additionally, both types of astrocytes express *Kcnj16* (encoding Kir5.1), and thus the ratio between the Kir4.1 homomer and the Kir4.1/Kir5.1 heteromer might play an important role in K^+^ uptake as well [36]. Finally, we also cannot exclude the possibility that 100 µM barium may not have been sufficient to fully block the K^+^ fluxes carried by Kir4.1 in brain slices that are 400 µm thick.

Similarly to K_2P_ channel inhibition, we found significant diversity between HR- and LR-astrocytes when chloride channels were inhibited using three different inhibitors. The inhibition of VRACs by tamoxifen and DCPIB led to a marked reduction of swelling in HR-astrocytes, which is in contrast to the proposed role of VRACs in RVD [37], [38]. Nevertheless, these channels have been shown to be responsible for chloride influx leading to persistent neuronal swelling during a prolonged excitotoxic insult [16]. On the other hand, in LR-astrocytes the application of Tamoxifen during OGD caused no volume changes. Nevertheless, when DCPIB, so far the most specific known inhibitor of VRACs, was applied, a significant increase in astrocyte volume was detected. We hypothesize that LR-astrocytes efficiently regulate their volume by VRACs, while HR-astrocytes might be more ischemia-affected cells, in which volume regulation is suppressed. As expected, the application of NPPB, a non-selective anion channel blocker, led to the inhibition of swelling in both HR- and LR-astrocytes during OGD, demonstrating that different chloride channel types might be responsible for Cl^−^ movement leading to astrocytic swelling during OGD. Since the molecular structure of VRACs has not yet been characterized [39], we performed gene expression profiling of anion/chloride channels possibly involved in astrocytic volume changes, such as plasmalemmal voltage-dependent anion channels (VDAC1-3) and chloride channels ClC2-10. Interestingly, we found marked differences in the expression levels of *Clcn2* (a gene for the ClC2 channel), which has been reported to be activated by cell swelling [39]. *Clcn2* is highly expressed only in astrocytic subpopulation 1 (comprising LR-astrocytes), thus possibly contributing to their increased ability to regulate their cell volume.

Since it has been demonstrated that some ion channels are co-expressed in certain regions of the astrocytic plasma membrane, such as the endfeet [8], [26], [27], [40], the importance of membrane protein interactions that might occur between different membrane units devoted to extracellular K^+^ buffering and water homeostasis should also be considered. Kir channels and AQP4 water channels are among the first candidates for such interactions (specifically Kir4.1), as demonstrated previously [41], [42]; however, regulatory volume decrease and volume homeostasis in astrocytes can also involve the co-localization/interaction of transient receptor potential vanilloid-4 (TRPV4) channels and AQP4 water channels occurring in a Ca^2+−^dependent manner, as demonstrated by Benfenati and co-authors [43]. Despite the fact that we found comparable *Aqp4* expression in both astrocytic subpopulations, our preliminary data also revealed marked differences in *Trpv4* levels in cortical astrocytes of EGFP/GFAP mice (unpublished data), a finding that accords well with a previously published study on primary cultures of cortical astrocytes in which only a certain subpopulation of astrocytes (∼60%) responded to a TRPV4 agonist or hypoosmotic stress [44]. However, further studies are necessary to clarify the role of TRPV4 channels in the diverse volume regulation of the two astrocytic subpopulations.

Finally, the inhibition of Na^+^-K^+^-Cl^−^ co-transporter also revealed differences between the two astrocytic populations. In HR-astrocytes we observed a marked reduction of swelling following bumetanide application during OGD, while the volume of LR-astrocytes remained unaffected. Our observations in HR-astrocytes are in agreement with those describing the involvement of NKCCs in astrocyte swelling and their contribution to cytotoxic edema [45]–[49]. The absence of a bumetanide effect on volume changes in LR-astrocytes might be explained by low NKCC protein levels, which were described by Yan and co-authors [50]. They found only a weak expression of NKCC1 in adult rat cortical astrocytes, while stronger expression was detected in perivascular astrocytes. Nevertheless, our PCR analysis did not reveal significant differences in the gene expression levels of *Nkcc1* among cortical astrocytes, and the overall expression levels were quite low in both astrocytic subpopulations.

No differences between LR- and HR-astrocytes were found during the inhibition of K^+^-Cl^−^ co-transporters or the Na^+^-dependent glutamate transporters EAAT1 (GLAST) and EAAT2 (GLT-1). KCC co-transporters contribute to the OGD-induced cell swelling in both subpopulations and accordingly no differences in the gene expression levels were found between subpopulation 1 and 2. The participation of KCCs in K^+^/Cl^−^ uptake under pathological conditions has been suggested previously in studies performed in the chicken retina [5]. Similarly, a block of glutamate co-transporters did not reveal differences between HR- and LR-astrocytes. In both HR- and LR-astrocytes, glutamate transporters contribute to the cell volume increase, which is in accordance with previous studies [51], [52].

We have proposed that differences in the extent of astrocytic swelling during OGD may be due to distinct ion channel and transporter expression. However, the possibility that HR- and LR- astrocytes face high/low [K^+^]_o_ and excitatory amino acid concentrations that could trigger distinct mechanisms of cell swelling and cell volume regulation has to be also considered. Although we have shown that their location in the cortical layers does not affect the extent of volume changes during OGD, we cannot rule out that the different volume regulation of astrocytes might partially correlate with their contact with different types of synapses or neurons with different sensitivity to ischemia. Since the application of inhibitors on acute brain slices unquestionably affects all cell types, we cannot overlook their inhibitory effect on neurons. As the inhibition of EAATs, KCCs, VRACs and K^+^ channels led to the reduction of HR-astrocyte swelling, we suggest that the effect of the inhibitors might be partially achieved by a block of K^+^ and EAA efflux from neurons, resulting in decreased astrocytic uptake.

Taken together, our results describe astrocytic subpopulations differing in their gene expression levels of K^+^ and Cl^−^ channels, which participate in astrocyte swelling as well as their volume regulation. To the best of our knowledge, this is the first study showing astrocyte heterogeneity in the cortex with respect to distinct expression profiles of K^+^ and Cl^−^ channels. Additionally, these findings support our earlier hypothesis that HR- and LR-astrocytes differentially regulate their volume during OGD based on their different expression levels of ion channels/transporters.

## Supporting Information

Figure S1 **3D-confocal morphometry of GFAP/EGFP astrocytes.** An EGFP-labeled astrocyte sectioned into a uniformly spaced (1 µm) set of 2D parallel images [29]. The cell surface was found in each image using an edge-detecting algorithm, and the area of the image surrounded by the edge was calculated for each image (bottom). For the cell soma volume (highlighted by full line) and the total cell volume (highlighted by dotted line) calculations, the area of interest was chosen for each individual cell.
(TIF)

Figure S2 **Scheme of the experimental sequence for measuring astrocyte volume changes during OGD.** Superimposed confocal images of an EGFP-labeled cortical astrocyte in ACSF, during 20-minute OGD and during a 20-minute co-application of ACSF_OGD_ and inhibitor. The volume changes were quantified every 10 minutes.
(TIF)

Figure S3 **Distribution of two astrocyte populations in the cortex of GFAP/EGFP mice related to gender or their location in the cortical layers.** **A:** Percent ratio of HR- (black)/LR- (white) astrocytes in the cerebral cortex of female (n = 193; left) and male (n = 168; right) mice. **B:** Percent ratio of HR-(black)/LR- (white) astrocytes in cortical layers I (n = 46), II–III (n = 113) and IV–VI (n = 133).
(TIF)

Table S1 **Sequences of primers used for quantitative single-cell PCR.** Genes used for analyses in experiments I and II are in bold.
(DOC)

Table S2 **Number of cells positive for individual genes – experiment I.**
(DOC)

Table S3 **Number of cells positive for individual genes – experiment II.**
(DOC)

Table S4 **Experiment I - Spearman correlation coefficients.** Significant correlations are in bold.
(DOC)

Table S5 **Experiment II - Spearman correlation coefficients.** Significant correlations are in bold.
(DOC)
