# Supplementary figures and images for: Distinct Expression/Function of Potassium and Chloride Channels Contributes to the Diverse Volume Regulation in Cortical Astrocytes of GFAP/EGFP Mice
Source: PLoS One. 2012 Jan 11;7(1):e29725. doi: 10.1371/journal.pone.0029725 (PMC3256164; doi:10.1371/journal.pone.0029725)

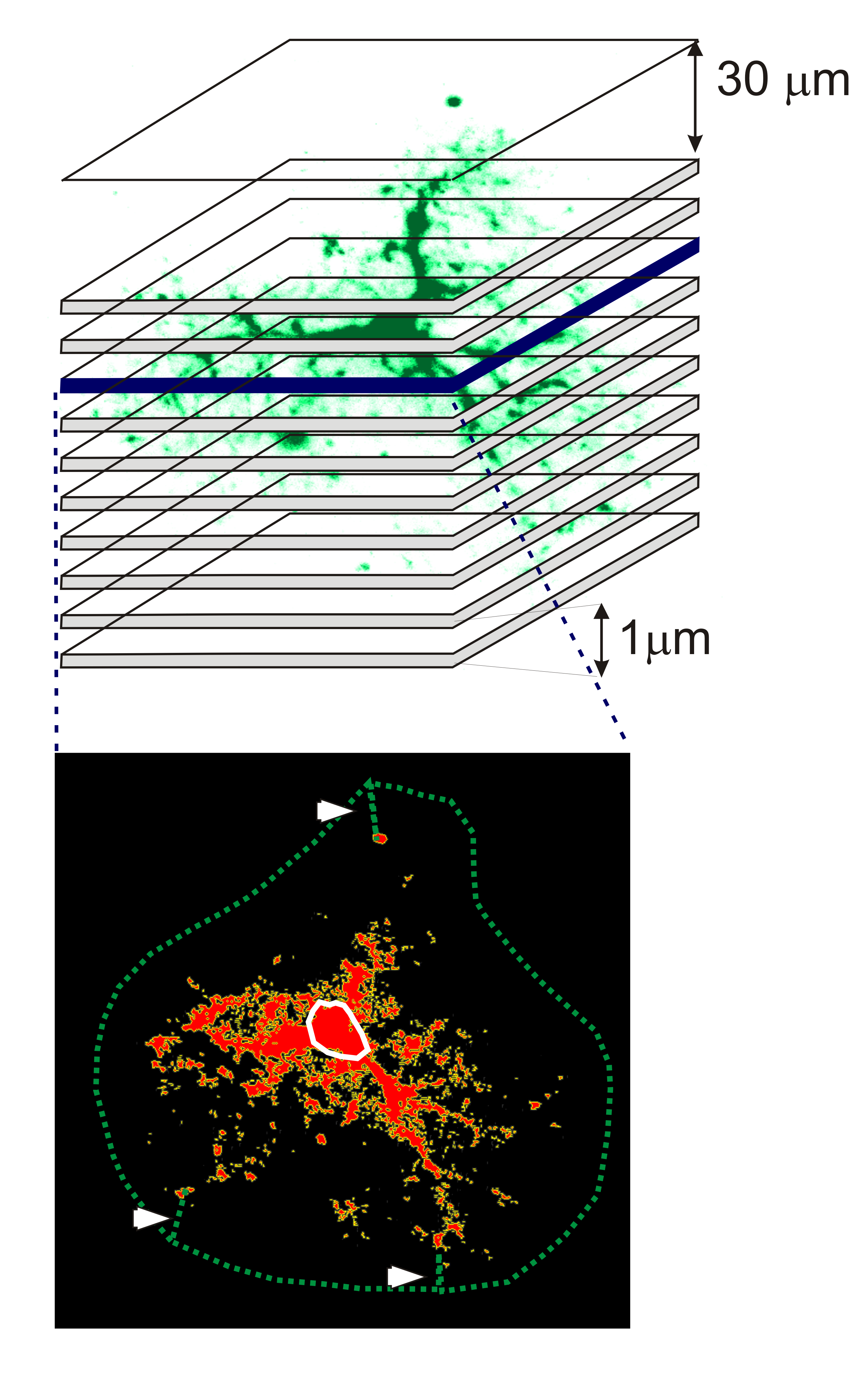

Supplement: Figure S1 — 3D-confocal morphometry of GFAP/EGFP astrocytes. An EGFP-labeled astrocyte sectioned into a uniformly spaced (1 µm) set of 2D parallel images [29]. The cell surface was found in each image using an edge-detecting algorithm, and the area of the image surrounded by the edge was calculated for each image (bottom). For the cell soma volume (highlighted by full line) and the total cell volume (highlighted by dotted line) calculations, the area of interest was chosen for each individual cell. (TIF) [file pone.0029725.s001.tif]

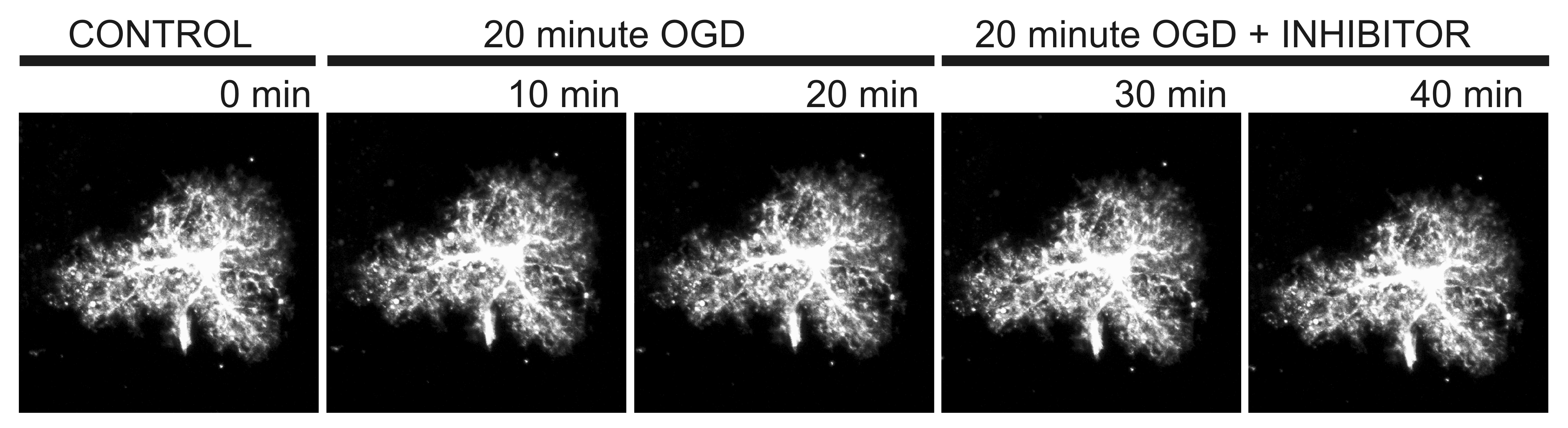

Supplement: Figure S2 — Scheme of the experimental sequence for measuring astrocyte volume changes during OGD. Superimposed confocal images of an EGFP-labeled cortical astrocyte in ACSF, during 20-minute OGD and during a 20-minute co-application of ACSFOGD and inhibitor. The volume changes were quantified every 10 minutes. (TIF) [file pone.0029725.s002.tif]

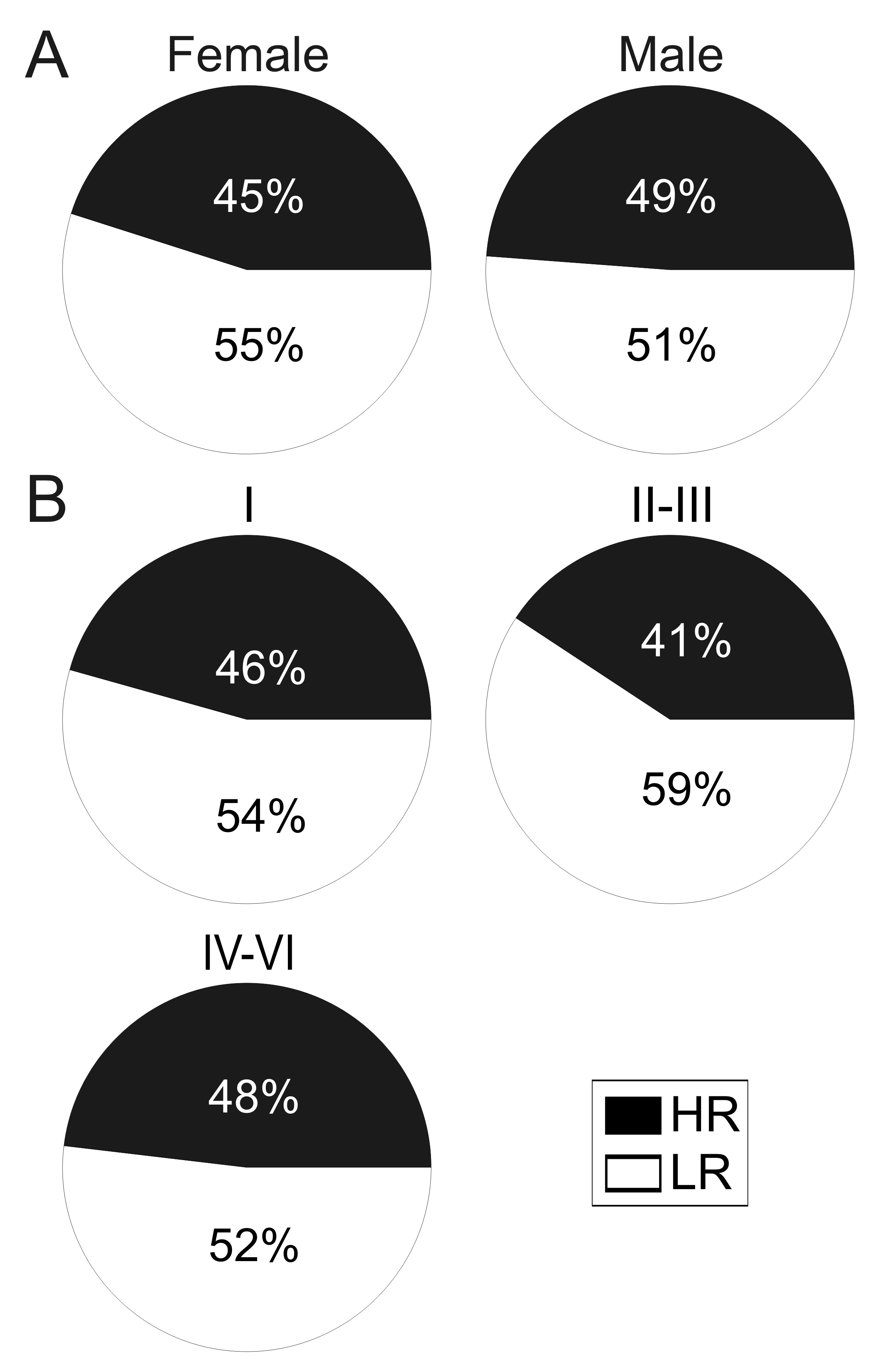

Supplement: Figure S3 — Distribution of two astrocyte populations in the cortex of GFAP/EGFP mice related to gender or their location in the cortical layers. A: Percent ratio of HR- (black)/LR- (white) astrocytes in the cerebral cortex of female (n = 193; left) and male (n = 168; right) mice. B: Percent ratio of HR-(black)/LR- (white) astrocytes in cortical layers I (n = 46), II–III (n = 113) and IV–VI (n = 133). (TIF) [file pone.0029725.s003.tif]
