# Supplementary material for: Distinct Expression/Function of Potassium and Chloride Channels Contributes to the Diverse Volume Regulation in Cortical Astrocytes of GFAP/EGFP Mice
Source: PLoS One. 2012 Jan 11;7(1):e29725. doi: 10.1371/journal.pone.0029725 (PMC3256164; doi:10.1371/journal.pone.0029725)
Supplement: Table S1 — Sequences of primers used for quantitative single-cell PCR. Genes used for analyses in experiments I and II are in bold. (DOC) [file pone.0029725.s004.doc]

**Table S1. Sequences of primers used for quantitative single-cell PCR**

| **Gene** | **PubMedID** | **Forward Primer** | **Length** | **Exon-exon spanning** | **Efficiency** | **Protein** |
| --- | --- | --- | --- | --- | --- | --- |
|  |  | **Reverse Primer** |  |  |  |  |
| ***Aqp4*** | NM_009700.2 | F: CGGCATCCTCTACCTGGTCACA | 82 | yes | 0.96 | AQP4 |
|  |  | R: GCCAGCGGTGAGGTTTCCAT |  |  |  |  |
| *Atp1a1* | NM_144900.1 | F: CCTCTGCTTCGTGGGTCTTATC | 128 | yes | 0.97 | ATPaseα1 |
|  |  | R: TCGCTGTGATTGGATGGTCTC |  |  |  |  |
| *Atp1a2* | NM_178405.3 | F: GAAGGACTTGGCTTGCTAA | 107 | no | 0.95 | ATPaseα2 |
|  |  | R: GCTACGAGGACGAGGATA |  |  |  |  |
| ***Clcn2*** | NM_009900.2 | F: TGCCAATGTCTTCCTTACTCTG | 198 | yes | 0.96 | ClC2 |
|  |  | R: ATTCGGTAGGTGCTGCTATC |  |  |  |  |
| *Clcn3* | NM_007711.3 | F: AATTGTTGACGATATTCCTGACC | 151 | yes | 0.98 | ClC3 |
|  |  | R: TGGGGATAAACAAGCCTGAC |  |  |  |  |
| *Clcn4* | NM_011334.3 | F: CCTCGTGCTCGTCGTATC | 127 | no | 0.98 | ClC4 |
|  |  | R: CCTCTTGCCTTCATTCTTGCTATA |  |  |  |  |
| *Clcn7* | NM_011930.3 | F: CATTGACATTGTAGTGGAGAACCT | 137 | yes | 0.98 | ClC7 |
|  |  | R: CACGAGTACGAAGGCAGAG |  |  |  |  |
| *Cspg* | NM_139001.2 | F: TGATGGAAGTGAGACACAGACAGA | 102 | no | 0.92 | CSPG4 |
|  |  | R: GGAAGGATGGTGATCGTGAAGG |  |  |  |  |
| ***Eaat1*** | NM_148938.3 | F: ATCGTCCTGCCTCTCCTCTAC | 159 | yes | 0.98 | EAAT1 |
|  |  | R: GTCCACACCATTGTTCTCTTCCA |  |  |  |  |
| ***Eaat2*** | NM_011393.2 | F: TCTTGGATGGAGGTCAGATTGT | 198 | yes | 0.95 | EAAT2 |
|  |  | R: CCACCACATTGACTGAAGTTCT |  |  |  |  |
| *Gfap* | NM_010277.3 | F: ACAGACTTTCTCCAACCTCCA | 159 | yes | 1 | GFAP |
|  |  | R: CAGGGCTCCATTTTCAATC |  |  |  |  |
| ***Kcc1*** | NM_009195.2 | F: TACAAGTACATCGAGTACCAAGG | 159 | yes | 0.95 | KCC1 |
|  |  | R: GTCTAACTTAAGCAGCACCAGGAG |  |  |  |  |
| *Kcnj1* | NM_019659.3 | F: ACATATTTGGGCGTTCTCGG | 93 | no | 1.02 | Kir1.1 |
|  |  | R: ACCTCGACTGTGCATCTACA |  |  |  |  |
| ***Kcnj10*** | NM_001039484.1 | F: AACTTGGGAGATTGAGATATGATATA | 129 | no | 0.94 | Kir4.1 |
|  |  | R: AAGTCTGAATACTTCCTTCTGTAC |  |  |  |  |
| ***Kcnj16*** | NM_010604.3 | F: CCTGTGTCTCCTCTTGAAGG | 158 | no | 0.93 | Kir5.1 |
|  |  | R: TGTGCTTAGGTGATACAATACGG |  |  |  |  |
| ***Kcnj2*** | NM_008425.4 | F: TCCCTCCCTTTCCCAAACAC | 134 | no | 0.99 | Kir2.1 |
|  |  | R: GAGGCTTGATTTTGAGACGC |  |  |  |  |
| *Kcnj3* | NM_004981.1 | F: ATGGACTAGATGACATTAGCACAA | 164 | no | 0.97 | Kir3.1 |
|  |  | R: AGGAACTGAACTTATTCGTTGGA |  |  |  |  |
| *Kcnj4* | NM_008426.1 | F: GACCCTCCTCGGACCTTAC | 150 | yes | 0.96 | Kir2.3 |
|  |  | R: CTGGCCGTTCTTCTTGACAA |  |  |  |  |
| *Kcnj8* | NM_008428.4 | F: CAAGTAAGGGCAGAGTAGAGAGAC | 187 | no | 0.96 | Kir6.1 |
|  |  | R: CGTGTGGTTCAATGGCTCAT |  |  |  |  |
| ***Kcnk1*** | NM_008430.2 | F: CGGTACGATATTCTGTTCCTGTAT | 77 | no | 0.87 | TWIK-1 |
|  |  | R: TCTCTTCCTCACTTGTTGTCTG |  |  |  |  |
| ***Kcnk10*** | NM_029911.4 | F: CACTGTGGCTATCTCCTTAACC | 111 | no | 0.95 | TREK-2 |
|  |  | R: GGCTGAGGCGGTGTAATC |  |  |  |  |
| ***Kcnk2*** | NM_010607.2 | F: GCATAGGTCGTCTCTAACAGTAG | 97 | no | 0.92 | TREK-1 |
|  |  | R: TGAATTACCAGCCTTCTCTTATCC |  |  |  |  |
| ***Nkcc1*** | NM_009194.3 | F: ATTACGGTGGCTAACACTGG | 98 | no | 0.94 | NKCC1 |
|  |  | R: GCATCTCTAAGAACTAATGGTTGA |  |  |  |  |
| *Pdgfra* | NM_011058.2 | F: AAGAGACCCTCCTTCTACCAC | 142 | yes | 0.97 | PDGFαR |
|  |  | R: TATCAGAGTCCACCCGCAT |  |  |  |  |
| *Taut* | NM_009320.4 | F: CTGAACCAGGAGAGGGCACTAC | 197 | no | 0.94 | TAUT |
|  |  | R: ACACAGGACAACAGGAACACAGT |  |  |  |  |
| ***Vdac1*** | NM_011694.4 | F: TCGCAGTTGGCTATAAGACG | 156 | yes | 0.95 | VDAC1 |
|  |  | R: TTCCGAAGCGAGTGTTACTG |  |  |  |  |
| ***Vdac2*** | NM_011695.2 | F: ACTTTCAGATAGCGGAGTGG | 156 | yes | 0.98 | VDAC2 |
|  |  | R: AGCATAGGGTGGAGGGATAC |  |  |  |  |
| ***Vdac3*** | NM_011696.1 | F: TGGGAGAATAAGTTGGCTGAAG | 124 | yes | 0.96 | VDAC3 |
|  |  | R: TACTGCCGAGACTAAAACAATCC |  |  |  |  |
