# Supplementary material for: Distinct Expression/Function of Potassium and Chloride Channels Contributes to the Diverse Volume Regulation in Cortical Astrocytes of GFAP/EGFP Mice
Source: PLoS One. 2012 Jan 11;7(1):e29725. doi: 10.1371/journal.pone.0029725 (PMC3256164; doi:10.1371/journal.pone.0029725)
Supplement: Table S2 — Number of cells positive for individual genes – experiment I. (DOC) [file pone.0029725.s005.doc]

**Table S2. Number of cells positive for individual genes – experiment I**

|  |  | ***Nkcc1*** | ***Eaat1*** | ***Vdac2*** | ***Aqp4*** | ***Eaat2*** | ***Vdac3*** | ***Clcn2*** | ***Vdac1*** | ***Kcc1*** |
| --- | --- | --- | --- | --- | --- | --- | --- | --- | --- | --- |
| **Astrocytes** | 103 | 11 | 99 | 69 | 92 | 102 | 32 | 33 | 35 | 31 |
|  |  | 10.7% | 96.1% | 67.0% | 89.3% | 99.0% | 31.1% | 32.0% | 34.0% | 30.1% |
| **Subpop. 1** | 72 | 3 | 69 | 48 | 67 | 72 | 21 | 2 | 24 | 22 |
|  |  | 4.2% | 95.8% | 66.7% | 93.1% | 100.0% | 29.2% | 2.8% | 33.3% | 30.6% |
| **Subpop. 2** | 31 | 8 | 30 | 21 | 25 | 30 | 11 | 31 | 11 | 9 |
|  |  | 25.8% | 96.8% | 67.7% | 80.6% | 96.8% | 35.5% | 100.0% | 35.5% | 29.0% |
