# Supplementary material for: Distinct Expression/Function of Potassium and Chloride Channels Contributes to the Diverse Volume Regulation in Cortical Astrocytes of GFAP/EGFP Mice
Source: PLoS One. 2012 Jan 11;7(1):e29725. doi: 10.1371/journal.pone.0029725 (PMC3256164; doi:10.1371/journal.pone.0029725)
Supplement: Table S3 — Number of cells positive for individual genes – experiment II. (DOC) [file pone.0029725.s006.doc]

**Table S3. Number of cells positive for individual genes – experiment II**

|  |  | ***Eaat1*** | ***Eaat2*** | ***Kcnj10*** | ***Kcnj16*** | ***Kcnk2*** | ***Clcn2*** | ***Kcnj2*** | ***Kcnk1*** | ***Kcnk10*** |
| --- | --- | --- | --- | --- | --- | --- | --- | --- | --- | --- |
| **Astrocytes** | 52 | 52 | 52 | 39 | 17 | 15 | 23 | 8 | 30 | 11 |
|  |  | 100.0% | 100.0% | 75.0% | 32.7% | 28.8% | 44.2% | 15.4% | 57.7% | 21.2% |
| **Subpop. 1** | 27 | 27 | 27 | 24 | 8 | 1 | 3 | 4 | 23 | 5 |
|  |  | 100.0% | 100.0% | 88.9% | 29.6% | 3.75 | 11.1% | 14.8% | 85.2% | 18.5% |
| **Subpop. 2** | 25 | 25 | 25 | 15 | 9 | 14 | 20 | 4 | 7 | 6 |
|  |  | 100.0% | 100.0% | 60.0% | 36.0% | 56.0% | 80.0% | 16.0% | 28.0% | 24.0% |
