# Supplementary material for: Distinct Expression/Function of Potassium and Chloride Channels Contributes to the Diverse Volume Regulation in Cortical Astrocytes of GFAP/EGFP Mice
Source: PLoS One. 2012 Jan 11;7(1):e29725. doi: 10.1371/journal.pone.0029725 (PMC3256164; doi:10.1371/journal.pone.0029725)
Supplement: Table S4 — Experiment I - Spearman correlation coefficients. Significant correlations are in bold. (DOC) [file pone.0029725.s007.doc]

**Table S4. Experiment I - Spearman correlation coefficients**

| ***Gene*** (Protein) | ***Nkcc1*** | ***Eaat1*** | ***Vdac2*** | ***Aqp4*** | ***Eaat2*** | ***Vdac3*** | ***Clcn2*** | ***Vdac1*** | ***Kcc1*** |
| --- | --- | --- | --- | --- | --- | --- | --- | --- | --- |
| ***Nkcc1*** (NKCC1) | 1.00000 |  |  |  |  |  |  |  |  |
| ***Eaat1*** (EAAT1) | 0.03245 p=0.7449 | 1.00000 |  |  |  |  |  |  |  |
| ***Vdac2*** (VDAC2) | -0.00141 p=0.9888 | **0.28649** p=**0.0033** | 1.00000 |  |  |  |  |  |  |
| ***Aqp4*** (AQP4) | -0.08017 p=0.4208 | **0.33249** p=**0.0006** | 0.05041 p=0.6131 | 1.00000 |  |  |  |  |  |
| ***Eaat2*** (EAAT2) | -0.05100 p=0.6089 | **0.33133** p=**0.0006** | **0.26354** p=**0.0072** | **0.32324** p=**0.0009** | 1.00000 |  |  |  |  |
| ***Vdac3*** (VDAC3) | 0.04606 p=0.6441 | **0.25208** p=**0.0102** | 0.06615 p=0.5068 | 0.08961 p=0.3680 | 0.14254 p=0.1509 | 1.00000 |  |  |  |
| ***Clcn2*** (ClC2) | 0.17434 p=0.0782 | -0.03806 p=0.7027 | -0.04120 p=0.6794 | -0.13070 p=0.1882 | -0.09945 p=0.3176 | 0.08392 p=0.3994 | 1.00000 |  |  |
| ***Vdac1*** (VDAC1) | 0.14476 p=0.1446 | **0.23226** p=**0.0182** | 0.14506 p=0.1437 | 0.07844 p=0.4309 | 0.10808 p=0.2772 | 0.03158 p=0.7515 | -0.04451 p=0.6553 | 1.00000 |  |
| ***Kcc1*** (KCC1) | -0.12862 p=0.1954 | 0.17333 p=0.0800 | 0.12945 p=0.1925 | -0.05685 p=0.5684 | 0.02871 p=0.7735 | 0.02780 p=0.7805 | -0.02703 p=0.7863 | -0.09689 p=0.3303 | 1.00000 |
