# Supplementary material for: Distinct Expression/Function of Potassium and Chloride Channels Contributes to the Diverse Volume Regulation in Cortical Astrocytes of GFAP/EGFP Mice
Source: PLoS One. 2012 Jan 11;7(1):e29725. doi: 10.1371/journal.pone.0029725 (PMC3256164; doi:10.1371/journal.pone.0029725)
Supplement: Table S5 — Experiment II - Spearman correlation coefficients. Significant correlations are in bold. (DOC) [file pone.0029725.s008.doc]

**Table S5. Experiment II - Spearman correlation coefficients**

| ***Gene*** (Protein) | ***Eaat1*** | ***Eaat2*** | ***Kcnj10*** | ***Kcnj16*** | ***Kcnk2*** | ***Cln2*** | ***Kcnj2*** | ***Kcnk1*** | ***Kcnk10*** |
| --- | --- | --- | --- | --- | --- | --- | --- | --- | --- |
| ***Eaat1*** (EAAT1) | 1.00000 |  |  |  |  |  |  |  |  |
| ***Eaat2*** (EAAT2) | **0.36683 p=0.0075** | 1.00000 |  |  |  |  |  |  |  |
| ***Kcnj10*** (Kir4.1) | 0.05740 p=0.6861 | **0.41680 p=0.0021** | 1.00000 |  |  |  |  |  |  |
| ***Kcnj16*** (Kir5.1) | 0.13389 p=0.3440 | -0.00799 p=0.9552 | 0.06450 p=0.6496 | 1.00000 |  |  |  |  |  |
| ***Kcnk2*** (TREK1) | -0.11501 p=0.4169 | -0.04916 p=0.7293 | -0.19955 p=0.1561 | 0.06413 p=0.6515 | 1.00000 |  |  |  |  |
| ***Cln2*** (ClC2) | -0.19786 p=0.1597 | -0.24017 p=0.0864 | -0.38298 p=0.0051 | 0.04364 p=0.7587 | 0.30157 p=0.0298 | 1.00000 |  |  |  |
| ***Kcnj2*** (Kir2.1) | 0.17218 p=0.2222 | -0.11029 p=0.4364 | 0.10532 p=0.4574 | 0.22847 p=0.1033 | 0.08159 p=0.5653 | -0.18779 p=0.1825 | 1.00000 |  |  |
| ***Kcnk1*** (TWIK1) | 0.09279 p=0.5130 | -0.02729 p=0.8477 | 0.24102 p=0.0852 | -0.07078 p=0.6181 | **-0.42969 p=0.0015** | **-0.48996 p=0.0002** | 0.10643 p=0.4527 | 1.00000 |  |
| ***Kcnk10*** (TREK2) | -0.08316 p=0.5578 | -0.04454 p=0.7539 | 0.16695 p=0.2368 | -0.00860 p=0.9517 | 0.08953 p=0.5279 | 0.00421 p=0.9764 | -0.10215 p=0.4711 | -0.18747 p=0.1832 | 1.00000 |
